# Health and Medical Sciences Productivity in the United Arab Emirates: A Bibliometric Analysis Based on 27 Years of Data From 1998 to Mid-2024

**DOI:** 10.7759/cureus.90629

**Published:** 2025-08-20

**Authors:** Hazem AbuEl-Enien, Mariam Al Harbi, Erik Koornneef, Alina Naeem, Shatha Jadallah, Kanmani Srinivasan, Nirmal Kumar M

**Affiliations:** 1 Content Development, PureHealth, Dubai, ARE; 2 Public Health Research, Director General Office, Abu Dhabi Public Health Center (ADPHC), Abu Dhabi, ARE; 3 Research, Innovation, and Education, PureHealth, Dubai, ARE; 4 Strategy Research and Innovation, PureHealth, Abu Dhabi, ARE; 5 Strategy, PureHealth, Dubai, ARE; 6 Medical and Patient Affairs, Leader Biotech Pharma, Dubai, ARE; 7 Operations and Management, Leader Biotech Pharma, Dubai, ARE

**Keywords:** bibliometric analysis, clinical research productivity, health sciences research, medical research uae, research collaboration, research trends in the uae (1998-2024)

## Abstract

The United Arab Emirates (UAE) has made significant strides in health-related research over the past decades. However, it is essential to monitor and review the status of health research to sustain progress. This study evaluates the UAE's health research landscape within the Gulf Cooperation Council (GCC) context and the broader Arab region. Bibliometric data on health research publications and clinical trials were reviewed from 1998 to mid-2024. Insights from regional analyses, including citation metrics, publication rates, and collaborative outputs, were integrated. Key challenges, such as funding limitations, regulatory barriers, and the decline in citation rates, were assessed to provide recommendations for improvement. The UAE has established itself as a key contributor to health research within the GCC, demonstrating significant progress in publication output and impact. While international collaborations have played a crucial role in enhancing visibility, there are areas for improvement, particularly in maintaining research influence and citation rates. To sustain progress, the UAE must prioritize high-impact collaborations, enhance research visibility, and invest in emerging areas like digital health and personalized medicine. Strategic reforms are essential to achieving a competitive health research ecosystem.

## Introduction and background

A country's research and development (R&D) depend on factors like social, political, and economic variables and the availability of research infrastructure in the shape of academic institutions, adequate healthcare systems, research funding, and trained researchers and support staff [1].

The United Arab Emirates (UAE) is ranked as a high-income country by the World Bank [2] and consists of seven independent states, or Emirates. Collectively, the UAE's federal government is embarking on a diversification program to reduce its economy's reliance on oil and transform it from a conventional, labor-intensive economy to one based on knowledge, technology, and skilled labor [3].

Recently, the UAE Ministry of Health and Prevention (MOHAP) and Elsevier published a report on the status of health research in the UAE from 2017 to 2022. In this report, MOHAP reviewed the UAE's performance in terms of health research over the years 2017-2022 in comparison with other Gulf Cooperation Council (GCC) countries (Bahrain, Kuwait, Iman, Qatar, Saudi Arabia, and UAE) and Group of 20 (G20) countries. This report outlined that UAE health research is growing three times the global rate. From 2019 to 2022, 27% of UAE research output is health-related, compared to the GCC health-related output of 32%, the G20 output of 37%, and the worldwide output of 36% [4].

**Figure 1:**
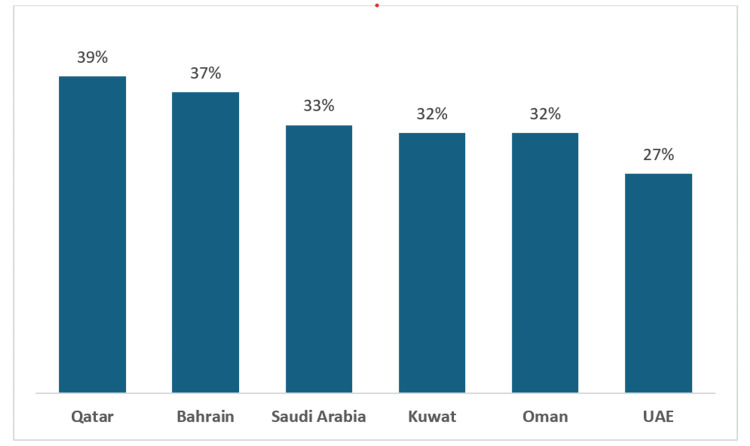
Health-related research output (%) from GCC Reference: [4] UAE: United Arab Emirates; GCC: Gulf Cooperation Council

UAE’s compound annual growth rate (CAGR) of health-related research is 25.2% from 2017 to 2022 [4]. Out of the 25.2%, around 80% of health-related research outputs are published articles with international collaborations. Internationally, GCC stands at around 32% of health-related research output, where Qatar has the highest health-related output at 39%, Bahrain at 37%, Saudi Arabia at 33%, Kuwait at 32%, Oman at 32%, and UAE at 27% (2019-2022) (Figure 1) [4].

The UAE is ranked second after the Kingdom of Saudi Arabia in conducting clinical trials among the GCC countries, reflecting its growing emphasis on advancing medical research and improving healthcare innovations [4]. This position highlights the UAE's commitment to fostering a robust research environment driven by investments in state-of-the-art facilities, regulatory frameworks, and strategic partnerships with global research institutions [4]. Despite its smaller population, the UAE has made significant strides in attracting and conducting clinical trials, positioning itself as a regional hub for cutting-edge healthcare R&D [4]. Compared to Saudi Arabia, which has 18896 health-related publications for 2022, the UAE’s health-related research production output is lower at 4345 publications for 2022. This difference can be explained by variables such as the UAE's small population size (nine million) compared to Saudi Arabia’s 35 million population [4].

The aim of our research was to explore and review health-related publication trends for the UAE, especially to understand the strengths and weaknesses of the research infrastructure and to shed light on what future steps can help increase health-related research quality and quantity and support the UAE in establishing a state-of-the-art health research hub.

## Review

Methodology

We extracted and analyzed the research output of the UAE in clinical research, health sciences, and medicine for the last 27 years, from 1998 to mid-year 2024 (at the time of this analysis), using the world's largest bibliometric database (Scopus/SciVal), which covers over 24000 research institutions and their associated researchers across more than 230 nations worldwide and encompasses over 40000 English-language journals in various disciplines of health and life sciences, physical sciences, and social sciences.

The search was carried out using the following research query: (“clinical research" OR "health sciences" OR ''medicine'' AND PUBYEAR > 1997 AND PUBYEAR < 2025 AND (LIMIT-TO (AFFILCOUNTRY, "United Arab Emirates")). This query ensured the retrieval of documents related to health, clinical, and medicine-related studies within the UAE from 1998 to mid-year 2024.

The documents collected in Scopus and the consideration of other metrics are based on the Research Metrics Guidebook and the Scopus Content Coverage Guide published by Elsevier [5-7].

We considered five raw quantity metrics, including I) documents per year, II) contributions by authors from UAE affiliations, III) the contributions of various UAE affiliations, IV) documents by subject area, and V) documents by publication type. These metrics can provide a comprehensive view of the quantity and nature of the research output from the UAE.

Quality of Research Output

In terms of quality measures of publications and their impact, we collected data on I) publications in top journal percentiles (1% and 10%), II) output in top citation percentiles (1%, 10%, field-weighted), III) output in top views percentiles (1%, 10%, field-weighted), IV) citation count, views count, and percentage of cited publications, V) field-weighted citation impacts (FWCIs), and VI) field-weighted views impacts (FWVIs).

Publications in top journal percentiles represent the number of publications in the top journals indexed by Scopus. Citations/views count refers to the total number of citations. An FWCI or FWVI of 1.0 indicates that the publications have been cited/viewed at the world average for publications in the same field.

International Collaborations and Academic-Corporate Collaborations

Since collaboration can positively affect research productivity and quality, we analyzed international and academic-corporate collaborations. This data was retrieved from SciVal and Scopus. We measured the extent of academic-corporate collaboration, the lack of academic-corporate collaboration, and the level of international collaboration. These metrics helped us understand the research's collaborative nature within and outside the UAE.

Research Disciplines

We examined research productivity across various health, clinical, and medicine-related 27 subject areas classified according to the All-Science Journal Classification (ASJC) from Scopus. The 10 key subject areas included medicine, biochemistry, genetics and molecular biology, pharmacology, toxicology and pharmaceutics, immunology and microbiology, neuroscience, agricultural and biological sciences, nursing, chemistry, multidisciplinary studies, social sciences, and other related fields. These subject areas provided insights into the focus and diversity of health-related research in the UAE.

This comprehensive methodology allows for a detailed assessment of the UAE’s research landscape in health, clinical, and medicine-related fields, guiding future research investments, policy development, and strategic planning to enhance the nation's scientific and healthcare capabilities.

Results

Quantitative Metrics

Documents per year: The overall number of documents published in the field of clinical research, health sciences, and medicine in the UAE from 1998 to mid-year 2024 totals 15267. This significant volume of publications reflects the country's growing contribution to the global research community over the past 27 years.

**Figure 2:**
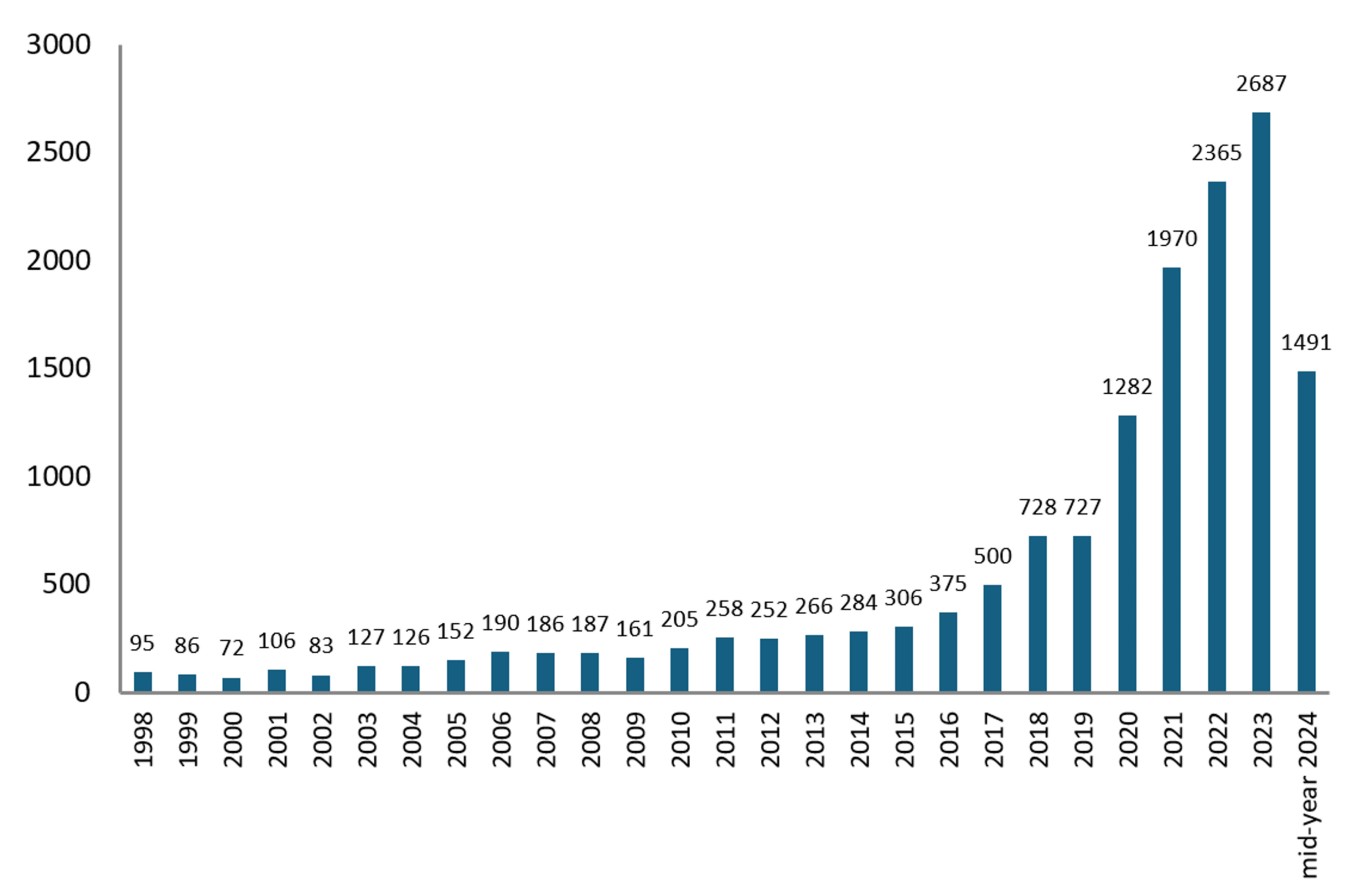
Health-related research publications per year

The data reveals a clear upward trend in the number of documents published per year. Starting from a modest 95 publications in 1998, the number of documents has seen a steady increase, reaching its peak in 2023 with 2687 publications. Notably, the years 2017 onwards show a particularly sharp rise in publication numbers, indicating an accelerated pace of research activity in recent years (Figure 2).

**Figure 3:**
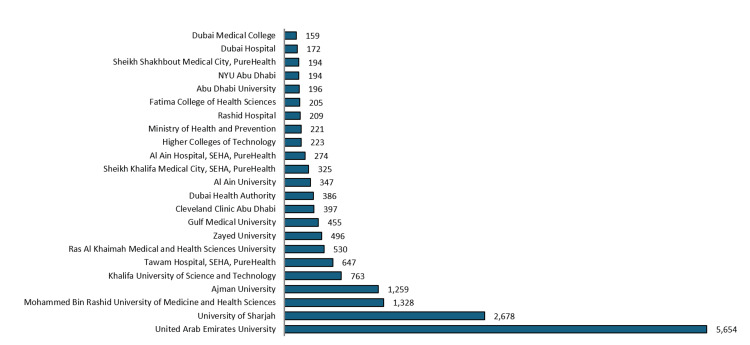
United Arab Emirates affiliation contributions

The UAE affiliations’ contributions: In terms of the number of health-related research publications per research institute, Figure 3 illustrates the contributions of several institutions, showing that the United Arab Emirates University stands out with the highest contribution of a total of 10,537 documents, where its College of Medicine and Health Sciences, United Arab Emirates University, contributes 4883 documents, indicating the significant portion of the research from the university is driven by its medical and health sciences faculty. The University of Sharjah and PureHealth Group (PHG) entities (Abu Dhabi Health Services Company- SEHA: (Tawam Hospital, Al Ain Hospital, Sheikh Khalifa Medical City (SKMC)/Abu Dhabi Health Research Center (ADHRC)) & Sheikh Shakhbout Medical City (SSMC)) also have substantial contributions, with 2678 and 1440 documents, respectively (Figure 3).

Documents by subject area: Medicine is the most significant area of research, accounting for over a third (5450 (35.70%)) of all publications. The second-largest category is biochemistry, genetics, and molecular biology (2229 (14.60%)), followed by social sciences (412 (2.70%)), chemistry (473 (3.1%)), nursing (473 (3.1%)), agricultural and biological sciences (489 (3.2%)), neuroscience (489 (3.2%)), immunology and microbiology (641 (4.2%)), along with pharmacology, toxicology, and pharmaceutics (1206 (7.9%)); collectively, these categories account for a total of 4580 (30%) documents. The "other" category, comprising 2977 (19.50%) of the publications, includes various fields such as computer science, dentistry, health professions, engineering, environmental science, and more. Though smaller in proportion, each of these areas contributes significantly to the holistic development of scientific knowledge and technological advancement.

Documents by publication type: Journal articles (11066 (72.50%)) are the most common type of publication, followed by reviews (2590 (17%)), which constitute the second portion of the publications, and afterward, various publication types are demonstrated, respectively, including book chapters (445 (2.9%)), conference papers (297 (1.9%)), letters (272 (1.8%)), editorials (260 (1.7%)), erratum (124 (0.8%)), note (121 (0.8%)), short surveys (40 (0.3%)), book (27 (0.2%)), and other types (25 (0.2%)). Each type serves a unique purpose, from sharing preliminary findings to providing expert commentary and comprehensive overviews.

Qualitative Metrics

**Figure 4:**
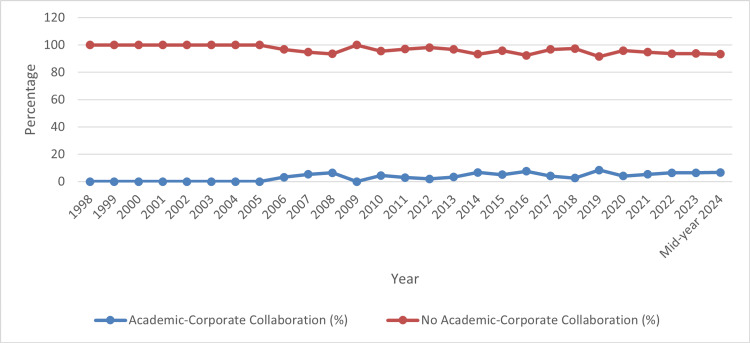
Publications yield with/without academic-corporate collaborations in the United Arab Emirates

Academic-corporate collaboration: Our study showed that publications yield without collaboration between academia and corporate entities (no academic-corporate collaboration) have consistently dominated the landscape, with an average of 94.7% over the observed period. The overall percentage of publications resulting from academic-corporate collaborations in clinical research, health sciences, and medicine in the UAE from 1998 to mid-year 2024 stands at a modest 5.3%. Figures 4 indicate that only a small fraction of publications involve partnerships between academic institutions and corporate entities, suggesting limited interaction between these sectors within the scope of the study.

Moreover, a detailed examination of the trends in academic-corporate collaborations across the UAE over a span of 27 years shows that there was no recorded academic-corporate collaboration, with the percentage remaining at 0% until 2005. This scenario changed slightly in 2006, with the collaboration percentage increasing to 3.2%. The highest recorded collaboration percentage was in 2016, at 7.6%. Post-2016, the data shows slight fluctuations, with the percentage hovering around an overall low trend.

**Figure 5:**
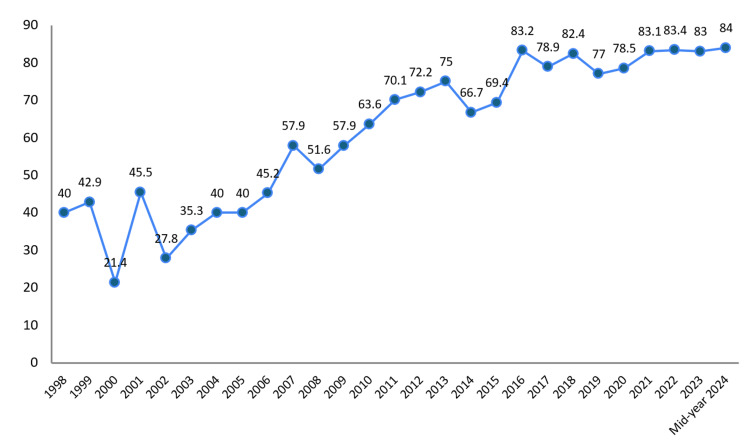
Distribution of the United Arab Emirates international collaboration (%)

Geographical collaboration (international collaboration (%)): Our analysis showed that the overall percentage of international collaborations in health-related fields in the UAE from 1998 to mid-year 2024 stands at 78% (11908). A detailed examination of the extent and trends of international collaborations in the UAE over a span of 27 years reveals significant growth. The percentage of international collaborations started at 40% (6107) in 1998, experienced a notable dip to 21.4% (3267) in 2000, but then saw a steady increase over the years. By 2013, the collaboration percentage had increased to 75% (11720), reaching its peak at 84% (13126) in mid-year 2024. This upward trend indicates a growing emphasis on international partnerships in health-related research in the UAE (Figure 5).

Views Metrics

View counts: The overall view count for health-related research publications in the UAE from 1998 to mid-year 2024 is 120061. This figure represents the total number of times these publications have been accessed or viewed, reflecting the interest and engagement of the global community with UAE-based health research outputs.

**Figure 6:**
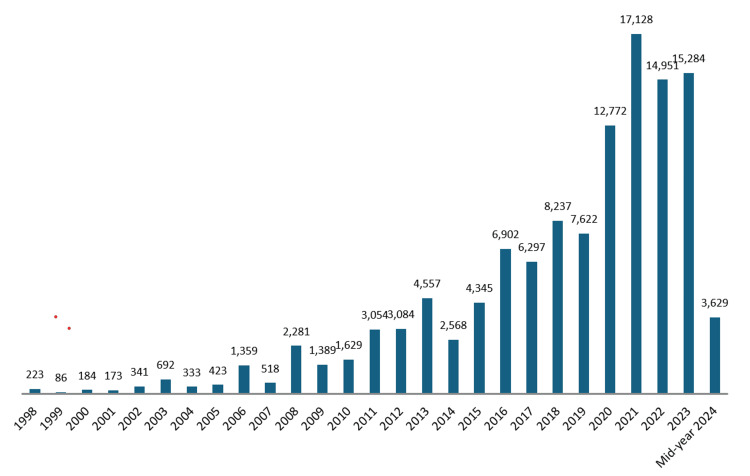
Year-wise view count

The view count for UAE health-related research publications shows a slightly fluctuating but generally increasing trend across 27 years (Figure 6). From 1998 to 2003, the view counts were relatively low in the early years, with counts such as 223 in 1998 and 341 in 2002. There was a noticeable increase starting in 2003 with 692 views, and this upward trend continued over the following years. Significant jumps were observed in 2011 (3054 views), 2013 (4557 views), and 2016 (6902 views). The highest recorded view counts occurred in the last five years of the study, with a peak in 2021 at 17128 views. This rise indicates a growing interest and increased visibility of UAE health research in recent years (Figure 6).

Output in top views percentiles (%): The overall percentage of UAE health-related publications in the top 1% and 10% by CiteScore Percentile stands at 3.9% and 23.9%, respectively. This indicates that many of the UAE's research outputs are among the most viewed globally, highlighting these publications' high visibility and impact.

The highest recorded percentage in the top 1% by CiteScore Percentile was in 2016, at 6.7%. With notable peaks in 2008, 2016, and 2021. While for the top 10% by CiteScore Percentile, the highest recorded percentage was in 2003, at 41.2%.

**Figure 7:**
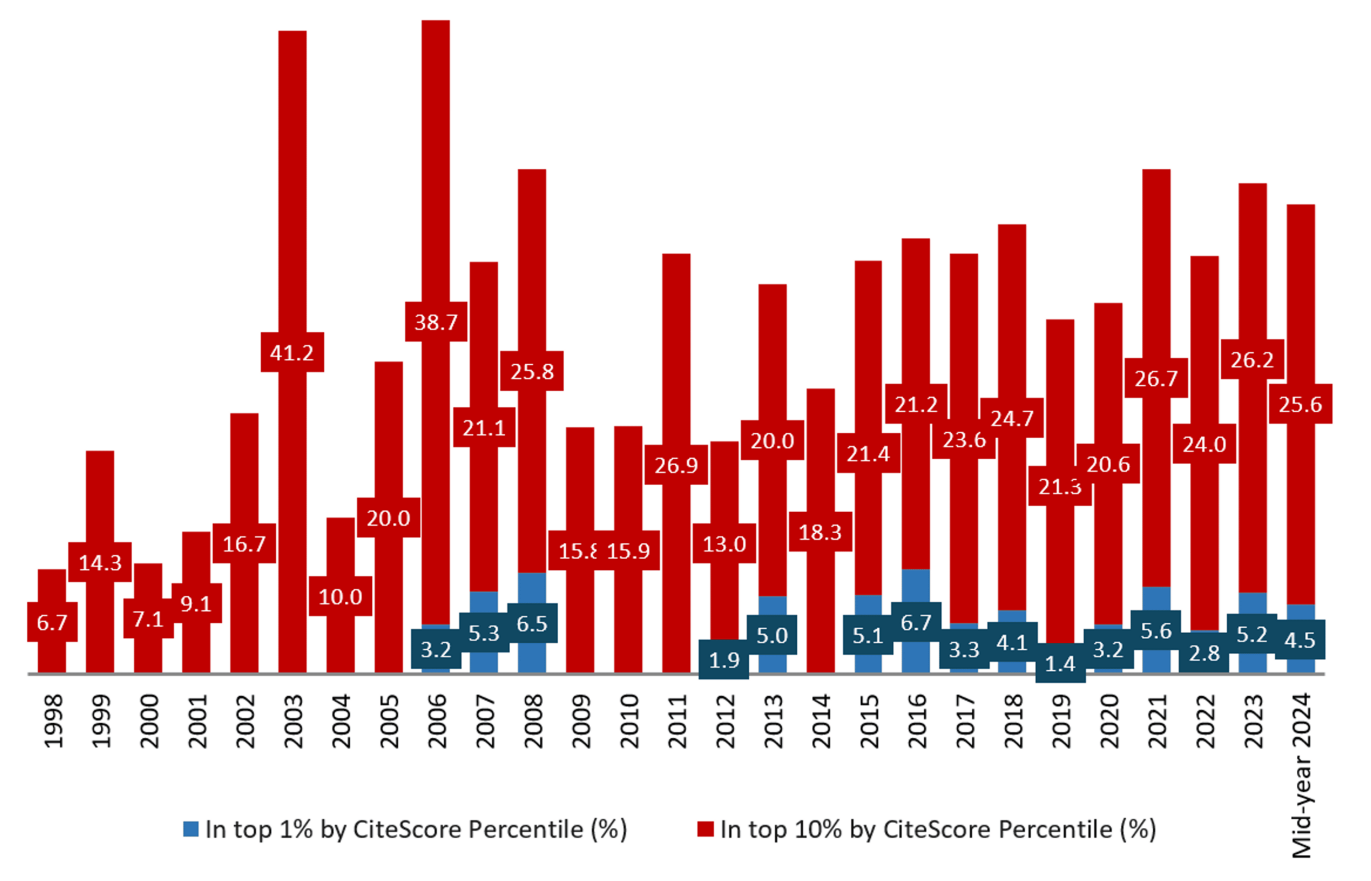
Output in top views percentiles

From 2018 onwards, the percentage of publications in the top 1% shows a steady presence, with slight fluctuations but generally maintaining a higher level compared to the earlier years. Meanwhile, the percentage in the top 10% shows a more pronounced increase. Since 2017, the values have remained consistently high, indicating an upward trend (Figure 7).

Field-weighted views impact: The FWVI is a metric that measures the impact of a publication relative to the global average in its field. An FWVI of 1.0 indicates that the publication has been viewed at the global average for similar publications.

The overall FWVI of publications in health-related fields in the UAE from 1998 to mid-year 2024 is 1.85. This indicates that, on average, publications from the UAE are viewed 85% more often than the global average for similar publications, suggesting relatively high visibility and interest in the research produced.

**Figure 8:**
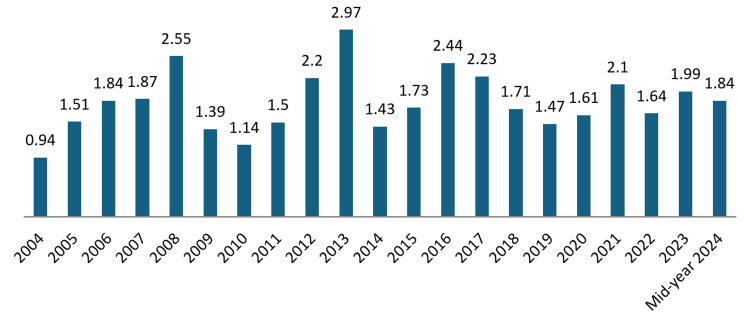
Field-weighted views impact (FWVI)

From 1998 to 2003, there were no recorded impacts (Figure 8), indicating either a lack of publications or insufficient data. In 2004, the FWVI was 0.94, rising steadily to 1.51 in 2005 and peaking at 2.55 in 2008. The FWVI saw a dip in 2009 to 1.39 but recovered over the following years, with notable peaks at 2.97 in 2013 and 2.44 in 2016. In recent years, the FWVI has stabilized around 1.84, with the lowest in 2019 at 1.47 and recovering to 1.84 in 2024. This trend demonstrates periods of high impact, reflecting varying levels of international interest and engagement with UAE health-related research.

Citation Metrics

Citation count: The overall citation counts for clinical research, health sciences, and medical sciences publications from the UAE between 1998 and mid-year 2024 stands at an impressive 74631. This high figure reflects the cumulative impact and recognition of research outputs from the UAE over this period.

**Figure 9:**
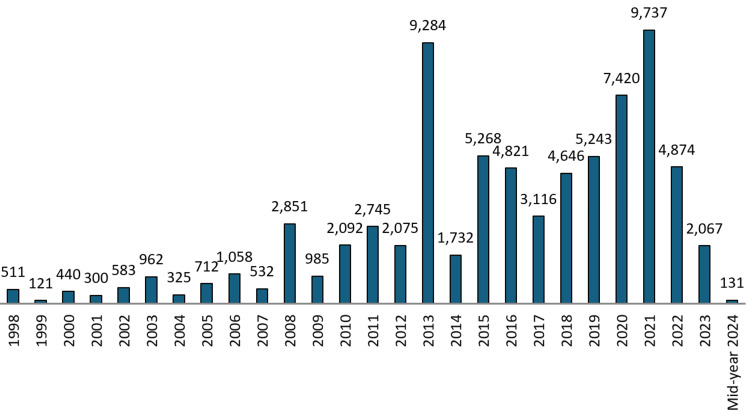
Citation count

The citation count trend from 1998 to 2024 shows significant fluctuations (Figure 9), with notable peaks and troughs. The early years (1998-2002) saw relatively low citation counts, with the lowest count recorded in 1999 (121 citations) and a gradual increase peaking in 2003 with 962 citations. The mid-period (2003-2014) is marked by a steady increase in citations, with notable peaks in 2008 (2851 citations) and 2010 (2092 citations). The highest peak during this period was in 2013, with 9284 citations. In recent years (2015-2021), the trend continued upward, with another notable peak in 2021 with 9737 citations, demonstrating growing recognition and impact. After 2022-2024, a decline has been observed.

Field-weighted citation impact: The FWVI is a metric that measures the impact of a publication relative to the global average in its field. An FWCI of 1.0 indicates that the publication has been cited at the global average for similar publications. An FWCI greater than 1.0 signifies an above-average impact, indicating that the publication has been cited more frequently than the global average. This metric provides a normalized measure of citation impact, allowing for meaningful comparisons across different fields of research.

**Figure 10:**
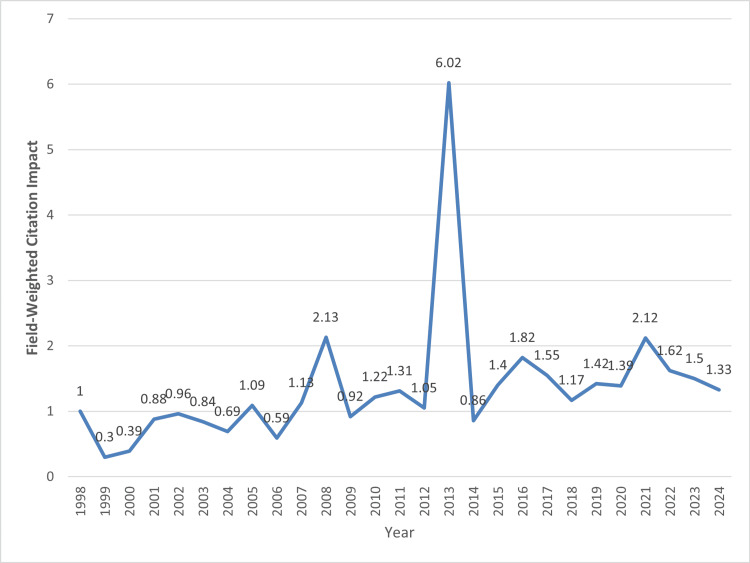
Distribution of field-weighted citation impact (FWCI)

The overall FWCI for publications in health-related fields in the UAE from 1998 to mid-year 2024 is 1.58 (Figure 10). This indicates that, on average, these publications have been cited 58% more frequently than the global average for similar publications, suggesting a relatively high impact of UAE research in these fields.

In recent years, although with a slight decline observed from 2022 to mid-year 2024, the FWCI values were 1.62, 1.5, and 1.33, respectively, still indicating a higher-than-average citation impact.

Output in top citation percentiles (field-weighted, %): The "output in top citation percentiles (field-weighted, %)" metric indicates the share of publications from the UAE that are among the most cited 1% and 10% publications worldwide. This metric is crucial for assessing the global impact and recognition of the UAE's research outputs, highlighting the most influential works within the academic community.

**Figure 11:**
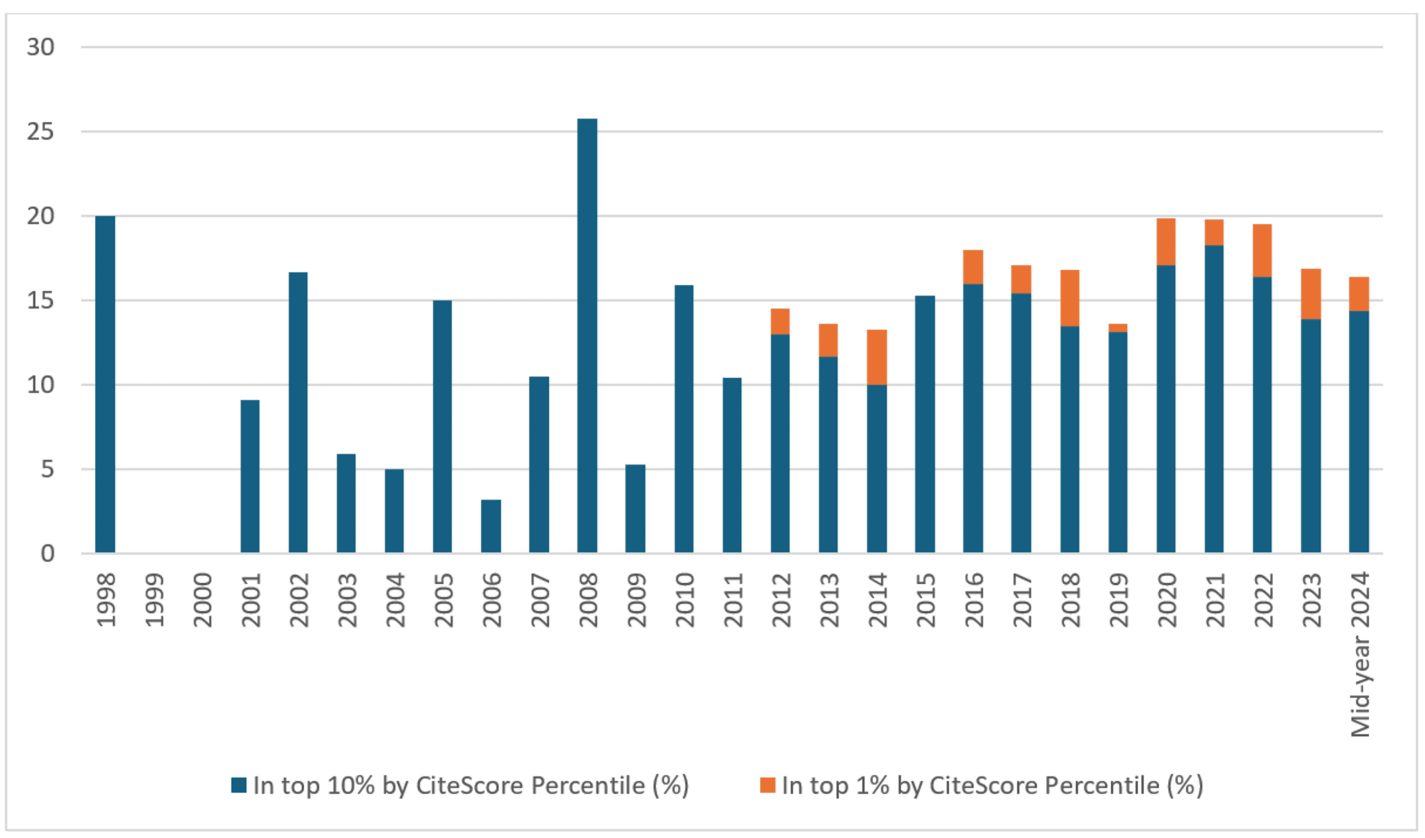
Output in top citation percentiles (field-weighted, %)

The overall analysis reveals that 14.9% of UAE publications fall within the top 10% worldwide by CiteScore, while 1.6% are among the most cited 1% of publications worldwide (Figure 11). In the top 10% category, recent years, particularly post-2015, showed a steady increase, with 18.3% in 2021 and 14.4% in mid-year 2024. While, in the top 1% category, the trend indicated a slight increase post-2012, reaching 3% in 2023.

Publications Metrics

Output in top citation percentiles (field-weighted, %): In 2024, the overall percentage of publications from the UAE in the top 10% of journals by CiteScore percentile reached 23%, while 2.4% of publications are in the top 1% by CiteScore percentile in 2024. These figures have fluctuated over the last decades but show a positive trend reflecting the quality and impact of research outputs from the UAE, indicating a significant portion of publications are in high-ranking journals.

**Figure 12:**
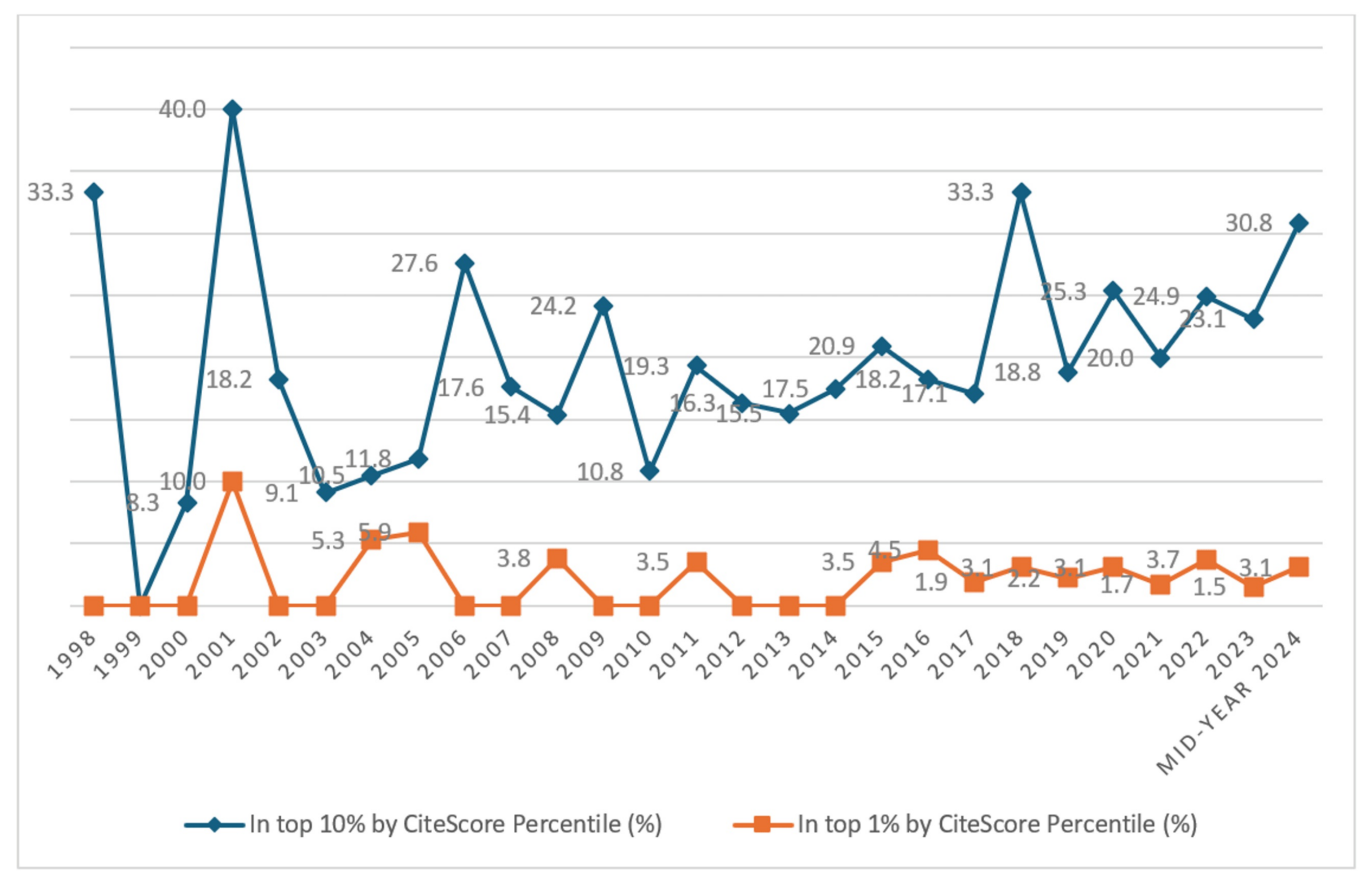
The United Arab Emirates’s publication in top journal percentiles

From 1998 to 2024, there have been fluctuations in the percentage of publications in the top 10% and top 1% of journals by CiteScore percentile; however, in the mid-year of 2024, there was an increasing trend in the top 10% and 1% with 30.8% and 3.1%, respectively (Figure 12).

Discussion

The UAE's position as the second-leading GCC country in conducting clinical trials highlights its commitment to advancing medical research and healthcare innovation. However, when contextualized within the broader Arab region, several gaps and opportunities emerge. For instance, research on clinical trials on oncology in Arab countries reveals a predominance of small-scale studies with limited external funding, particularly in trials involving only Arab countries [8]. In contrast, studies with joint Arab and non-Arab authorship tend to receive more funding and achieve higher impact factors [8]. The UAE's strong international collaboration, with 80% of health-related research outputs published in partnership with international institutions, positions it as a model for enhancing research quality and global visibility. Encouraging further alliances, especially in underrepresented fields such as oncology and precision medicine, could significantly elevate the UAE's research impact [8,9].

This bibliometric analysis presents a comprehensive assessment of the UAE's research output in clinical research, health sciences, and medicine over the 27 years from 1998 to mid-2024. To the best of our knowledge, this study is the first to evaluate the quantity and quality of UAE-based research in these domains, providing a foundation to understand the evolving trends and contributions of the country’s academic landscape across almost three decades of scientific work. It also offers insights into the UAE’s integration into the global scientific community through collaborative research efforts.

This analysis utilized Scopus and SciVal, two leading bibliometric databases, to ensure a complete and accurate representation of UAE-affiliated publications across different languages, including English and Arabic. The study examined multiple metrics, including publication volume, author contributions, institutional affiliations, and research subject areas. Quality metrics such as citations, FWVI, and visibility indicators - like views and output - in top journal percentiles were incorporated to evaluate the scholarly impact of UAE research. This dual approach captures the depth and breadth of research productivity, offering insights into the UAE’s ability to align its scientific output with international standards.

Research Growth and Trends

The UAE's relatively strong institutional framework has likely contributed to its notable research growth, as indicated by its CAGR of 25.5% in health-related publications from 2017 to 2022. However, challenges such as the decline in citation rates since 2022 may indicate potential issues in research dissemination or a focus on lower-impact publications. Addressing these challenges through targeted policies, such as incentivizing high-quality research and strengthening academic-corporate collaborations, could mitigate these declines and sustain growth.

The study reveals a consistent upward trend in research output, with significant growth observed from 2017 onward, reflecting the impact of recent policies and increased investment in R&D. The establishment of new research institutions/centers, such as ADHRC-SKMC and Khalifa University of Science and Technology in Abu Dhabi, and collaborations between academia, healthcare sectors, and international partners have significantly contributed to this expansion. Government initiatives focusing on healthcare and biomedical innovation, such as targeted funding programs and grants, have also been pivotal in fostering a research-conducive environment.

Much of the UAE's research output has been driven by UAE University and its College of Medicine and Health Sciences, which play leading roles in academic productivity and public health policy influence. Other institutions, such as the University of Sharjah, PHG entities (Abu Dhabi Health Services Company - SEHA: (Tawam Hospital, Al Ain Hospital, SKMC/ADHRC, and SSMC)) in Abu Dhabi, Mohammed Bin Rashid University of Medicine and Health Sciences in Dubai, and Gulf Medical University in Ajman, and Khalifa University in Abu Dhabi also contribute significantly, reflecting the growing diversity of health research institutions in the UAE (Figure 3).

Finally, the observed decline in the percentage of cited publications between 2022 and mid-2024, from 81% to 25.2%, warrants attention. This trend may reflect shifts in research priorities, changes in citation practices, or an overemphasis on quantity over quality. Future efforts should focus on aligning research output with global scientific priorities, promoting open-access dissemination, and addressing barriers to citation, such as limited international engagement in specific fields. Expanding research into emerging areas like artificial intelligence, digital health, and personalized medicine could enhance the UAE's standing as a global leader in health research.

When compared to other Arab countries, the UAE exhibits a more rapid acceleration in research productivity but also mirrors regional challenges related to funding, impact, and visibility. For instance, a bibliometric study on rheumatology research in Arab countries from 1975 to 2019 indicated a concentration of outputs in only a few countries, with the UAE ranking lower than Saudi Arabia, Egypt, and Tunisia in total output [10]. Another analysis of rehabilitation research in the Arab world highlighted a slow growth pattern and a general lack of inter-institutional collaboration, challenges that are partially addressed in the UAE but still persist [11].

Compared to Egypt and Saudi Arabia’s focus on clinical research in infectious diseases and oncology, the UAE's profile is more collaboration-driven, with a larger percentage of internationally co-authored papers [12]. However, its FWVI still lags behind leading countries in the region, as observed in studies analyzing bibliometric trends in neurology and spinal surgery [13,14].

Distribution Across Subject Areas

Our analysis shows that the dominant subject area is medicine, followed by biochemistry, genetics, and molecular biology. This aligns with the UAE's strategic focus on improving healthcare outcomes, developing advanced treatments, and addressing public health challenges. Research in areas like engineering, materials science, and chemistry, particularly with applications in health, highlights the UAE’s commitment to leveraging technology to advance medical sciences [9,15]. Additionally, while social sciences publications are relatively modest, their role is crucial for understanding healthcare systems and patient behavior and shaping health policies, reflecting a holistic approach to healthcare improvement [9,15].

The COVID-19 pandemic significantly influenced research trends in the UAE, leading to a surge in publications related to virology, public health, and epidemiology. Researchers focused on vaccine development, treatment protocols, and the socio-economic impact of the pandemic, increasing interdisciplinary studies. A bibliometric assessment revealed that UAE-affiliated authors contributed to a substantial number of COVID-19-related papers, with the top five journals publishing 93 such articles, accounting for 8.6% of the nation's total publications [16]. This surge underscores the UAE's commitment to addressing the pandemic through scientific inquiry and highlights the country's active role in global health research collaborations.

In addition to the overall volume, a thematic breakdown reveals that public health, infectious diseases, and internal medicine have dominated the UAE’s health research portfolio in the last five years. Notably, a growing number of studies in oncology, diabetes, cardiovascular diseases, and mental health have been observed. A recent review of UAE-based publications found that non-communicable diseases, particularly diabetes and cardiovascular diseases, were among the most frequently researched areas, reflecting the country's national health priorities. Preventive medicine and health promotion have also gained traction, especially in the wake of increased health awareness campaigns and national screening initiatives [10]. However, mental health and geriatric care remain underrepresented despite their rising relevance in the UAE's healthcare discourse, suggesting an opportunity for more targeted research in these areas [17].

The prominence of interdisciplinary research, with contributions spanning multiple subject areas, indicates the UAE's interest in tackling complex health challenges. This multidisciplinary focus promotes innovative solutions by integrating biomedical research with technological and social sciences perspectives, enhancing the potential for impactful outcomes [9,15].

Types of Publications and Scholarly Communication

The analysis of publication types highlights that the majority of research outputs are whole articles, indicating the UAE's focus on producing original scientific knowledge. A significant number of review articles demonstrate efforts to synthesize existing knowledge, address research gaps, and provide directions for future studies. This aligns with regional trends, as studies have shown that full articles dominate the research output of GCC countries, particularly in the health sciences, reflecting efforts to contribute to evidence-based practices and foundational research [9,17].

Furthermore, the notable review articles demonstrate the UAE's commitment to synthesizing existing knowledge and identifying research gaps. This approach is critical in guiding future studies and providing a comprehensive understanding of health-related challenges, as emphasized in regional bibliometric analyses [9].

Including other publication types, such as conference papers, letters, and editorials, illustrates the diverse methods UAE researchers employ to disseminate findings, share preliminary results, and engage with the scientific community. These formats are critical for fostering early-stage research collaborations and providing expert commentary on emerging issues in healthcare and beyond [9,15].

Collaboration Patterns and Strategic Gaps

The study reveals a strong trend toward international collaboration, with a growing number of publications co-authored with researchers from around the world. These collaborations enhance the visibility and impact of UAE research, allowing it to contribute meaningfully to global scientific knowledge. By fostering partnerships with leading research institutions worldwide, the UAE ensures that its research aligns with global healthcare priorities and scientific advancements. Papers with international collaborations achieve higher citations and are more likely to be published in high-impact journals [15,17]. The UAE's health-related outputs benefit significantly from international collaborations (80%), which sets a strong example within the GCC. However, fostering partnerships in underrepresented fields, such as oncology and non-communicable diseases, would be advantageous in enhancing the impact further [9].

However, the analysis also identified a notable gap in academic-corporate collaborations. The low proportion of joint publications between academic institutions and corporate entities indicates the need for targeted initiatives to strengthen these partnerships. Enhanced collaboration between academia and industry can foster innovation, translate research into practical applications, and address real-world healthcare challenges. Initiatives such as innovation hubs, joint research funding, and corporate-academia exchange programs could significantly enhance resource sharing and knowledge transfer, benefiting both sectors.

Scholarly Impact and Visibility

The quality of UAE research is reflected in its high citation counts, consistent presence in top journal percentiles, and growing visibility, as evidenced by increasing view counts over the years. The inclusion of publications in the top 10% and 1% of journals by CiteScore percentile underscores the international recognition and impact of UAE-based research. Despite fluctuations in citation metrics in recent years, the UAE has maintained a higher-than-average FWVI, indicating that its research is highly regarded within the global academic community.

The steady rise in views and publications in top citation percentiles reflects an increasing interest in UAE research outputs, particularly in the later years of the study period. This trend suggests that recent research efforts are not only meeting global quality standards but also attracting the attention of a wider audience. However, the observed decline in citation counts and the percentage of cited publications from 2022 to mid-2024 point to the need for more effective dissemination strategies to maintain and enhance the impact of current research outputs.

Current Challenges

Challenges emerging markets face in conducting clinical research, including regulatory barriers and limited funding, are also relevant to the UAE. Studies suggest that addressing these challenges through streamlined approval processes and support for early-phase trials could enhance the UAE’s competitiveness as a clinical trial hub [9,16]. Additionally, increased investment in research infrastructure and initiatives aimed at improving the quality of publications could mitigate the decline in citation rates observed in recent years.

Comparative insights from the Middle East emphasize the need for the UAE to align its research priorities with global trends. As measured by normalized citation metrics, the Middle East's research impact lags behind that of global leaders like Europe and North America [9,18]. To address this, UAE research institutions should prioritize publications in top-tier journals and engage in high-visibility global research initiatives. Strengthening interdisciplinary collaborations and focusing on emerging areas such as digital health and personalized medicine could further elevate the UAE’s standing on the global research stage.

Limitations

While this study provides valuable insights, several limitations should be acknowledged. First, although the use of Scopus and SciVal ensures comprehensive data collection, some UAE-affiliated research may not be captured, particularly publications in non-indexed journals or specialized regional databases. Moreover, PHG is a vast network of 11 healthcare entities throughout the UAE, with Abu Dhabi Health Services Company - SEHA as a PHG entity alone having 14 hospitals under it. We anticipate that this publication number will be much higher once all affiliated entity publication statistics are combined. Discrepancies in affiliation reporting practices and our current review scope of publication data from SCOPUS and SciVal are limitations in the reported outcome measures for reporting PHG publication statistics.

Second, the focus on bibliometric metrics does not capture the research's broader societal or real clinical impact. Future studies could incorporate additional qualitative assessments to evaluate how research outcomes translate into healthcare improvements and policy changes.

Finally, the data analyzed only extends to mid-2024, meaning that very recent trends, emerging research fields, or the impact of newly implemented policies throughout 2024 have not been fully captured. Future research should consider expanding the dataset and incorporating complete 2024 data.

## Conclusions

This bibliometric analysis offers a comprehensive overview of the UAE's health, medicine, and clinical sciences research landscape over the last 27 years. It highlights significant progress in research productivity, impact, international collaboration, and the increasing visibility of UAE research outputs. While the growing international partnerships underscore the UAE's commitment to contributing to global scientific knowledge, the low level of academic-corporate collaboration presents an area for strategic improvement. Strengthening these collaborations through targeted initiatives will be essential for translating research findings into practical healthcare solutions. The advancement in research and substantial government investment in medical research have significantly improved healthcare outcomes in the UAE, leading to enhanced disease prevention, better treatment options, and a more significant healthcare infrastructure.

Maintaining and improving the upward trajectory of research productivity and quality will require sustained investment, strategic dissemination efforts, and fostering interdisciplinary and collaborative research. By addressing the identified gaps and building on its strengths, the UAE can continue to enhance its research impact, contribute to global health solutions, and solidify its position as a leading player in the global scientific community.
